# Risk Factors for Anthracycline-Induced Cardiotoxicity

**DOI:** 10.3389/fcvm.2021.736854

**Published:** 2021-09-29

**Authors:** Shuo Qiu, Tian Zhou, Bo Qiu, Yuxin Zhang, Yonggang Zhou, Huihui Yu, Jingyi Zhang, Li Liu, Lijun Yuan, Guodong Yang, Yunyou Duan, Changyang Xing

**Affiliations:** ^1^Department of Ultrasound Diagnostics, Tangdu Hospital, Air Force Medical University, Xi'an, China; ^2^Department of General Surgery, Tangdu Hospital, Air Force Medical University, Xi'an, China; ^3^School of Nursing, Air Force Medical University, Xi'an, China; ^4^Department of Hematology, Tangdu Hospital, Air Force Medical University, Xi'an, China; ^5^Department of Biochemistry and Molecular Biology, Air Force Medical University, Xi'an, China

**Keywords:** anthracycline, hypertension, diabetes mellitus, obesity, chemotherapy, cardiotoxicity, global longitudinal strain

## Abstract

**Background:** Several cardiovascular risk factors have been suggested to be associated with anthracycline-induced cardiotoxicity, but their quantitative effects have not reached a consensus.

**Methods:** We searched PubMed, EMBASE, and Cochrane Library databases for manuscripts published from inception to February 2021, which reported the results of cardiotoxicity due to anthracycline chemotherapy without trastuzumab. Cardiotoxicity defined by any reduction of left ventricular eject fraction (LVEF) to below 50% or a >10% reduction from baseline was defined as the primary endpoint. Odd ratios (OR) with 95% confidence intervals (CI) were calculated using a random-effects model meta-analysis.

**Results:** A total of 7,488 patients receiving anthracycline chemotherapy without trastuzumab were included, who had at least one risk factor at baseline. Hypertension (OR: 1.99; 95% CI: 1.43–2.76), diabetes mellitus (OR: 1.74; 95% CI: 1.11–2.74), and obesity (OR: 1.72; 95% CI: 1.13–2.61) were associated with increased risk of cardiotoxicity. In addition, the relative reduction of global longitudinal strain (GLS) from baseline after anthracycline treatment could significantly improve the detection ability of cardiotoxicity (28.5%, 95% CI: 22.1–35.8% vs. 16.4%, 95% CI: 13.4–19.9%) compared with LVEF. The early detection rate of anthracycline-induced cardiotoxicity (3 months after chemotherapy) by GLS was 30.2% (95% CI: 24.9–36.1%), which is similar with the overall result of GLS.

**Conclusions:** Hypertension, diabetes mellitus, and obesity are associated with increased risk of anthracycline-induced cardiotoxicity, which indicates that corresponding protective strategies should be used during and after anthracycline treatment. The findings of higher detection rate and better early detection ability for cardiotoxicity than LVEF added new proofs for the advantages of GLS in detection of AIC.

## Introduction

In recent years, due to advances in cancer treatment and early detection strategies, the mortality rates of cancer have significantly declined (1). Studies have shown that in the next 10 years, long-term cancer survivors are expected to increase by about 30% (2). However, the improvement in survival rate comes with an increased risk of cardiotoxicity (3). Anthracyclines, a vital component of numerous cytotoxic regimens, is a broadly used chemotherapy for many malignancies including breast cancer, sarcoma, lymphoma, bladder cancer, and some leukemias (4). For anthracyclines, such as doxorubicin, when the cumulative dose is 250 to 400 mg/m^2^, there will be 6 to 33% of patients with reduced left ventricular ejection fractions (LVEF) (5). Cardiotoxicity and subsequent heart failure caused by anthracyclines are the most common clinical symptoms in patients with breast cancer, lymphoma, and hematopoietic cancers (6).

Pre-existing cardiovascular risk factors have been suggested to be associated with increased incidences of anthracycline-induced cardiotoxicity (AIC). It was reported that the incidence of cardiotoxicity in patients with hypertension, diabetes mellitus, or obesity is remarkably higher than that in normal patients (7). However, the results of the quantitative risk effects of those cardiovascular factors vary between different studies. It was mainly attributed to the confounding treatment of other chemotherapy and/or cardioprotective drugs and different definitions of cardiotoxicity (8–11).

Echocardiography is the cornerstone for cardiac imaging in patients with chemotherapy, with advantages of wide availability, easy repeatability, and free from radiation exposure. The new onset of heart failure symptoms, a 10% asymptomatic drop in LVEF or a drop in LVEF from baseline to <55% determined by two-dimensional (2D) echocardiography after cancer treatment, are considered as cardiotoxicity (12). However, the low sensitivity of LVEF for the detection of subclinical changes in cardiac function may cause delayed diagnosis of AIC (13). With the development of echocardiography technology, speckle tracking echocardiography (STE) has been widely used in early and sensitive detection of the subclinical myocardial impairment, among which the most commonly used method is global longitudinal strain (GLS). A recent meta-analysis showed that GLS has a good prognostic performance for the development of cardiotoxicity in patients receiving anthracyclines (14). The early detection value of GLS in AIC is of great interest, which may change the current LVEF-guided AIC treatment.

We conducted a meta-analysis to assess the risk factors associated with AIC and explore the early detection ability of GLS for AIC.

## Methods

### Search Strategy

A systematic review was performed, adhering to Preferred Reporting Items for Systematic Reviews and Meta-Analyses (PRISMA) guidelines (15) (Supplementary Table 1). We searched PubMed, EMBASE, and Cochrane Library databases for manuscripts published from inception to February 2021. Key words included in the search were anthracycline, doxorubicin, chemotherapy, cancer therapy, cardiotoxicity, cardiomyopathy, risk factors, hypertension, diabetes mellitus, obesity, overweight, hypercholesterinemia, and smoking. In addition, we have expanded the scope of our search to manually search the list of references from original studies, gray literature, and records, regardless of language and publication years.

### Study Selection and Quality Assessment

Two reviewers (SQ and TZ) independently searched the titles and abstracts of the above databases by using a standardized form. Any discrepancies between the two reviewers were resolved by consensus. Eligible studies were identified if they met the following inclusion criteria: (1) adult participants ≥18 years of age; (2) research types, both retrospective studies, case–control study, and clinical randomized controlled trials; (3) LVEF or GLS was assessed at baseline and at the end of treatment; and 4) follow-up duration ≥3 months. Studies were mainly excluded for the following reasons: (1) the type of study is not appropriate, including reviews, editorials, and case report; (2) published in a format other than English; (3) LVEF data were incomplete; and (4) animal studies. For multiple manuscripts published from the same cohort, the meta-analysis included the most recent analysis and the highest number of results. In addition, for studies including patients with trastuzumab, we only extract the data from the group of patients without trastuzumab. The methodological quality of included studies was assessed by the revised Quality Assessment of Diagnostic Accuracy Studies (QUADAS-2) tool. This tool comprises four domains: patient selection, index test, reference standard, and flow and timing. Each domain is assessed in terms of risk of bias, and the first three domains are also assessed in terms of concerns regarding applicability (16).

### Data Extraction and Clinical End Points

The literature search, study selection, study appraisal, and data extraction were pre-defined in the protocol and were independently conducted by two investigators (SQ and TZ). Data were extracted and included study setting, cohort descriptors, prevalence of cardiotoxicity, baseline and follow-up data for LVEF, and diagnostic criteria. Any discrepancies were resolved by consensus.

The primary clinical endpoint was defined as any reduction of LVEF to below 50% or a >10% reduction from baseline. Another endpoint is GLS, which is currently used as an indicator in 2D STE parameters to predict cardiotoxicity (13, 17–19). In previous studies, patients with cardiotoxicity were defined as GLS with an average reduction of 10–20% (20). This study defines cardiotoxicity as relative percentage reduction of ≥10% in GLS from baseline.

### Statistical Analysis

The summary incidence of AIC was calculated from the number of patients receiving chemotherapy based on anthracycline in retrospective cohort studies, case–control study, and clinical randomized controlled trials. In order to calculate the odds ratio (OR) and 95% confidence interval (CI) of each risk factor, data on the number of patients with various risk factors in the cardiotoxicity group and the non-cardiotoxicity group were extracted.

The pooled estimates were derived using the DerSimonian and Laird random-effect model. Heterogeneity among studies was assessed using Cochran's *Q* test and by measuring consistency (*I*^2^). To assess the potential impact of publication bias, we checked the symmetry of the funnel chart. Sensitivity analyses were performed by comparing the conclusion after removing the study with the largest proportion with the original one. Statistical analyses were performed with Review Manager 5.3 (version 5.3.5, Copenhagen: The Nordic Cochrane Center, The Cochrane Collaboration) and Comprehensive Meta-Analysis Version 3.0 software (Biostat, Englewood, NJ), and *P* < 0.05 was considered statistically significant.

## Results

### Study Selection and Baseline Characteristics

**Figure 1:**
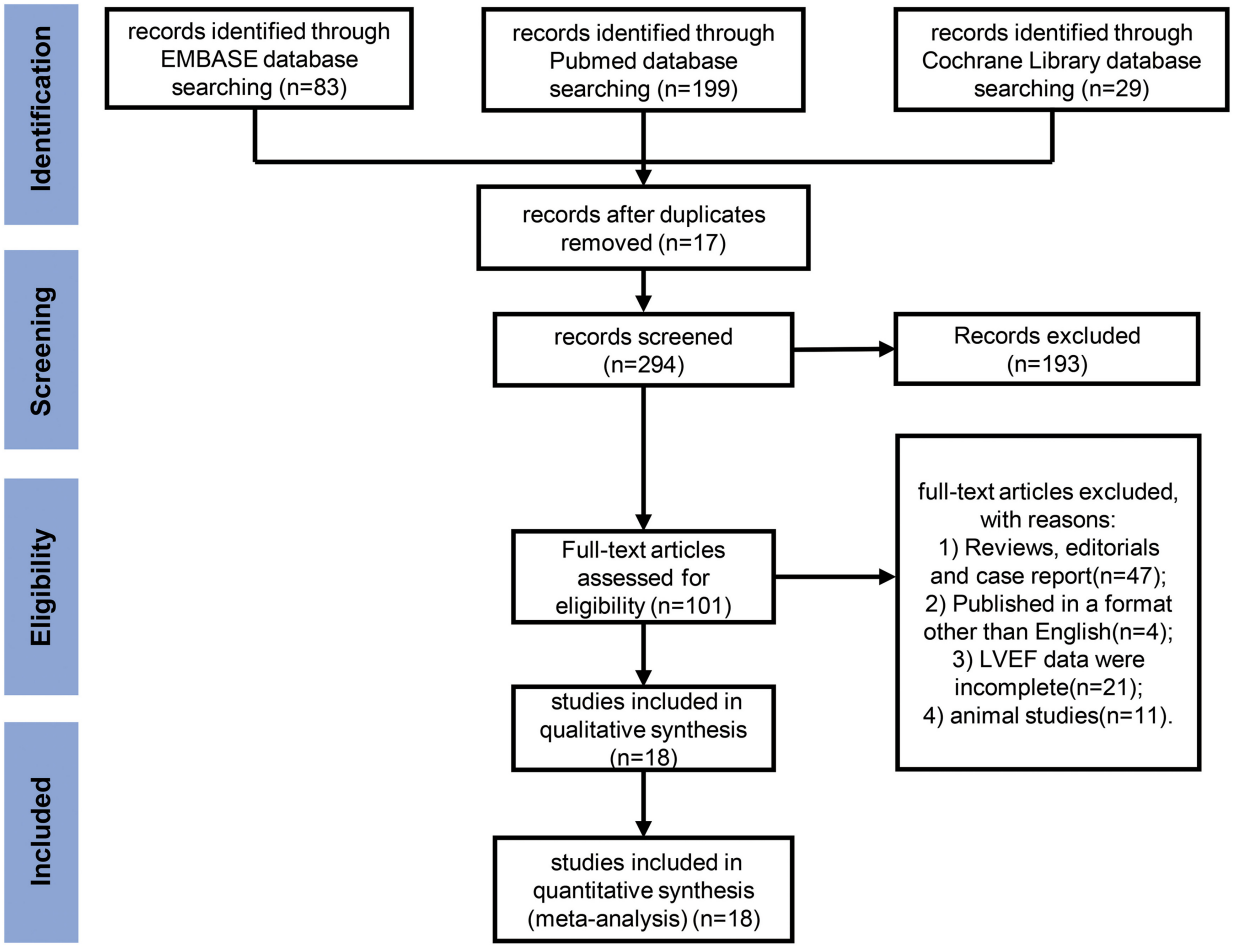
Preferred Reporting Items for Systematic Reviews and Meta-Analyses (PRISMA) flow diagram for meta-analysis. A flow diagram of the studies screened, assessed for eligibility, and included in the review, with reasons for exclusions.

The process of study selection is presented in a PRISMA flow diagram (Figure 1). According to the inclusion and exclusion criteria, a total of 18 studies (21–38) were included for analysis. The detailed characteristics of these studies, including treatment regimens, patient population, primary endpoint, and number of patients are shown in Table 1. The total number of various types of cancer patients treated with anthracycline in the 18 studies was 7,488. Furthermore, 66.7% (12 studies) of the included studies were breast cancer. In terms of various risk factors analyses, 12 studies were included for hypertension, 8 studies for diabetes mellitus, 5 studies for obesity, 3 studies for overweight, 5 studies for hypercholesterinemia, and 9 studies for smoking. Moreover, the detection ability of LVEF and GLS for AIC with corresponding endpoints was analyzed in six studies.

**Table 1:** Characteristics of the studies and patients included in the meta-analysis.

**Reference**	**Study**	**Patient population**	**Anthracycline therapy**	**Primary endpoint**	**Patient numbers**
Vaitiekus et al. (21)	Retrospective	Breast cancer	Doxorubicin	LVEF decreased more than 10% from baseline	73
Chung et al. (22)	Retrospective	Breast cancer	Doxorubicin	LVEF decreases more than 10% from baseline or the LVEF declines under 55%	174
Cho et al. (23)	Retrospective	Breast cancer	Doxorubicin	LVEF decreases more than 10% from baseline or the LVEF declines under 55%	613
Hequet et al. (24)	Retrospective	Lymphoma	Doxorubicin	LVEF declines under 53% and FS declines under 25%	141
Wang et al. (25)	Retrospective	Cancer underwent anthracycline chemotherapy	Doxorubicin, idarubicin, and epirubicin	New York Heart Association grade III or IV congestive HF and cardiac arrest	2,285
Araujo et al. (26)	Retrospective	Breast cancer and hematologic cancer	Doxorubicin	LVEF decreases more than 10% from baseline or the LVEF declines under 50%	188
Piotrowski et al. (27)	Retrospective	Breast cancer	Doxorubicin	LVEF decreases more than 10% or 15% from baseline or the LVEF declines under 50%	253
Gunaldi et al. (28)	Retrospective	Breast cancer	Doxorubicin and epirubicin	LVEF decreases more than 10% from baseline or the LVEF declines under 50%	111
Cardinale et al. (29)	Retrospective	Cancer underwent anthracycline chemotherapy	Doxorubicin and epirubicin	LVEF decreases more than 10% from baseline or the LVEF declines under 50%	2,625
Toufan et al. (30)	Prospective	Breast cancer	Doxorubicin	LVEF decreases more than 10% from baseline; GLS decreases more than 15% from baseline	63
Kang et al. (31)	Retrospective	Large B-cell non-Hodgkin lymphoma	Epirubicin	LVEF decreases more than 10% from baseline; GLS decreases more than 15% from baseline	75
Sawaya et al. (32)	Prospective	Breast cancer	Doxorubicin and epirubicin	LVEF decreases more than 10% from baseline; GLS decreases more than 10% from baseline	43
Tang et al. (33)	Retrospective	Breast cancer	Doxorubicin	LVEF decreases more than 10% from baseline; GLS decreases more than 13.84% from baseline	86
Arciniegas et al. (34)	Retrospective	Breast cancer	Doxorubicin and epirubicin	LVEF decreases more than 10% from baseline; GLS decreases more than 15% from baseline	66
Aitelhaj et al. (35)	Retrospective	Breast cancer	Doxorubicin	LVEF decreases more than 10% from baseline or the LVEF declines under 50% or any symptoms or signs of heart failure	100
Caram et al. (36)	Retrospective	Breast cancer	Doxorubicin	The LVEF declines under 50%	165
Karanth et al. (37)	Retrospective	Breast cancer, lymphomas, gastrointestinal cancers, and sarcomas	Doxorubicin and epirubicin	The LVEF declines under 55%	217
Mina et al. (38)	Retrospective	Breast cancer	Doxorubicin and epirubicin	The LVEF declines under 50%	210

### Quality Assessment

**Figure 2:**
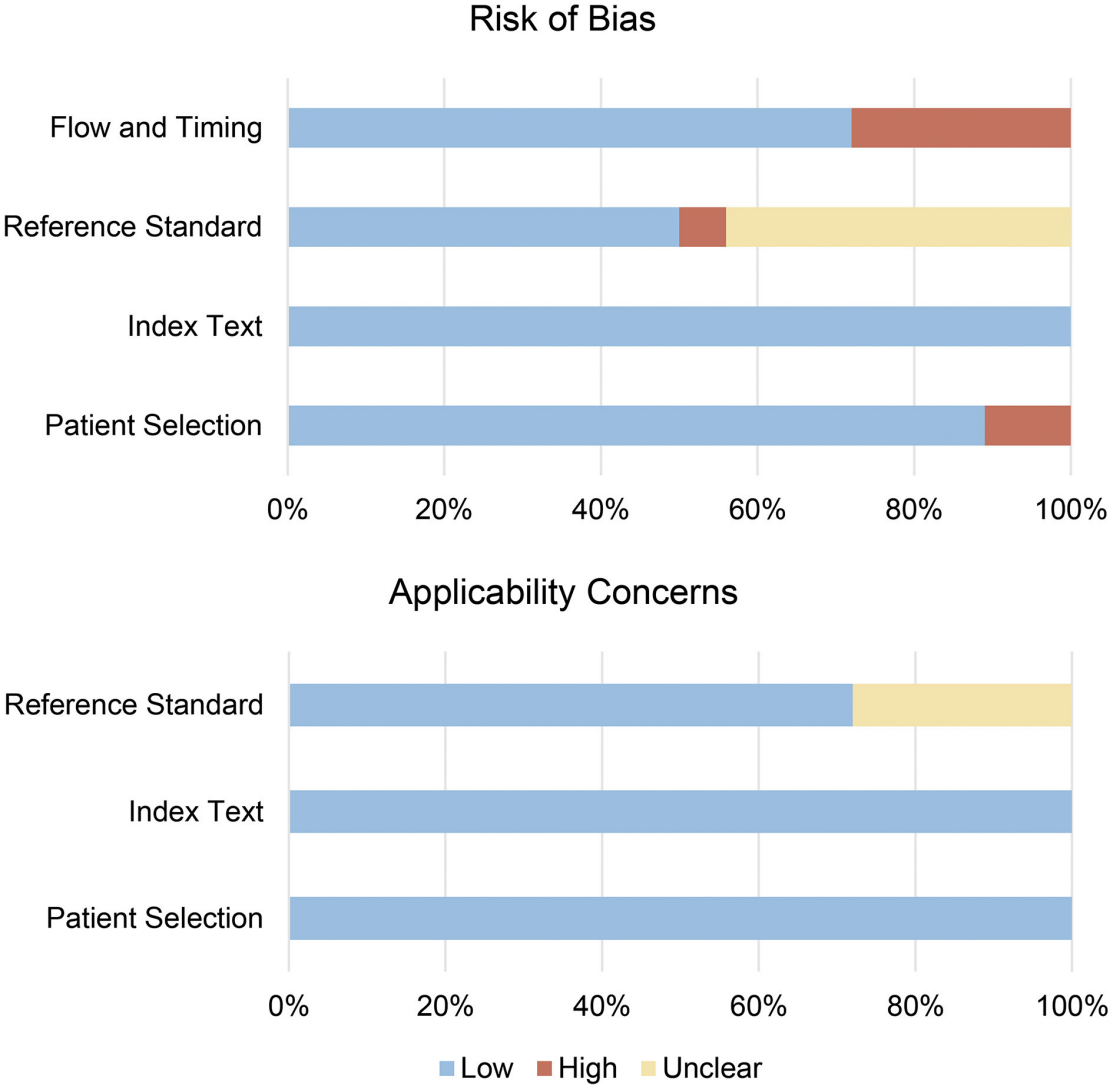
Risk of bias summary for included studies. The methodological quality of included studies was assessed with the revised Quality Assessment of Diagnostic Accuracy Studies (QUADAS-2) tool. This tool comprises four domains: patient selection, index test, reference standard, and flow and timing. Each domain is assessed in terms of risk of bias, and the first three domains are also assessed in terms of concerns regarding applicability.

As assessed by QUADAS-2, the bias risk mainly came from reference standard and flow and timing domains (Figure 2, Supplementary Table 2, and Supplementary Figure 1). Nine studies (50%) did not explain whether blinding was used in the interpretation of the results, for which only “unclear” or “high risk” was given for reference standard domain. Cases of five studies (27.8%) are not judged by a gold standard and thus evaluated as “high risk” for flow and timing domain. Additionally, two studies (11.1%) did not explain whether patients were included in a continuous or random way and are thus “high risk” for patient selection domain. Overall, the included studies were able to meet the appropriate quality standards.

### Incidence of AIC

**Figure 3:**
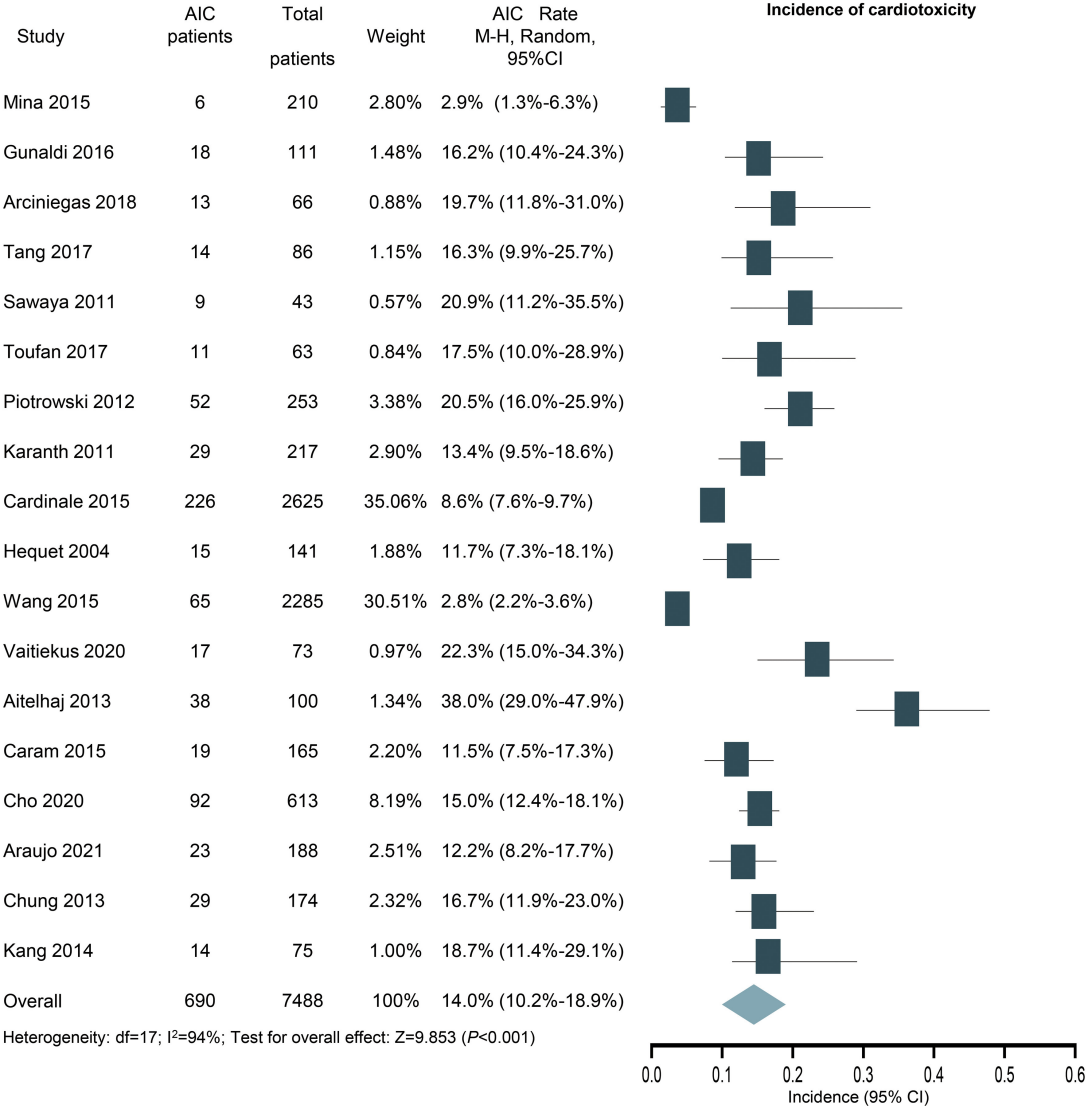
Forest plot of the incidence of anthracycline-induced cardiotoxicity. CI, confidence intervals; OR, odds ratio.

According to the primary clinical endpoint of LVEF, 690 out of the 7,488 patients were assessed as cardiotoxicity, resulting in an overall incidence of 14.0% (95% CI: 10.2 to 18.9%) for cardiotoxicity with high heterogeneity (*I*^2^ = 94%) (Figure 3). The result was still similar when the study contributing the largest number of patients was excluded (14.4%; 95% CI: 10.0–20.4%; *P* < 0.001).

### Risk Factors of AIC

#### Hypertension

**Figure 4:**
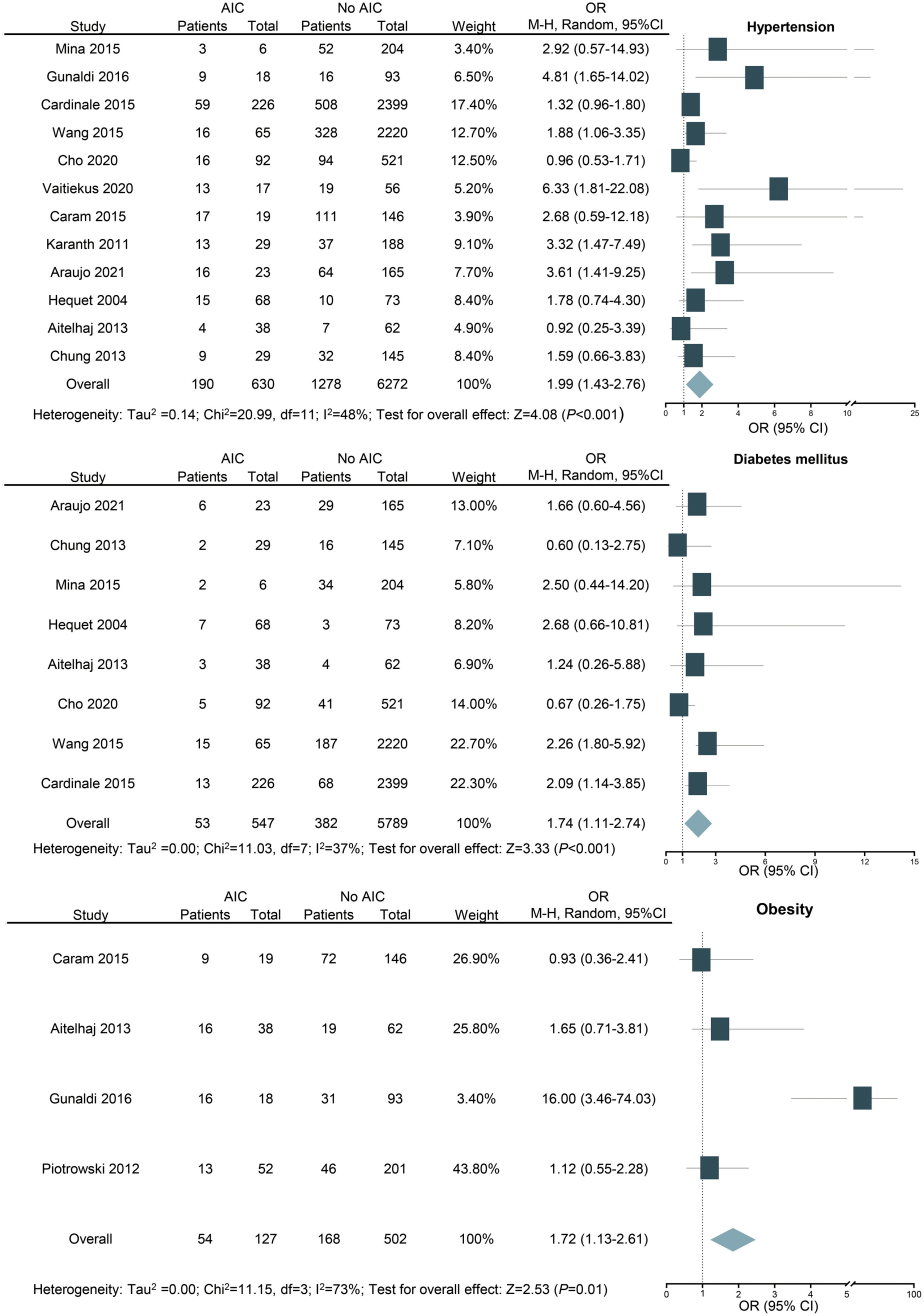
Forest plot for the effects of hypertension, diabetes mellitus, and obesity on the risk of anthracycline-induced cardiotoxicity. CI, confidence intervals; OR, odd ratios.

For the assessment of hypertension as a potential risk factor, 12 studies with 6,902 patients were included. Hypertension is identified as a risk factor for cardiotoxicity (OR: 1.99; 95% CI: 1.43–2.76; *P* < 0.001; Figure 4) with medium heterogeneity (*I*^2^ = 48%). In addition, the result was still similar when the study with the largest number of patients was excluded (OR: 2.18; 95% CI: 1.50–3.15; *P* < 0.001).

#### Diabetes Mellitus

For the assessment of diabetes mellitus as a potential risk factor, eight studies with 6,336 patients were included. Diabetes mellitus is revealed as a risk factor for AIC (OR: 1.74; 95% CI: 1.11–2.74; *P* < 0.001; Figure 4) with medium heterogeneity (*I*^2^ = 37%). Among the studies we included, there are three studies with fewer than five people with diabetes mellitus, which may be one of the sources of heterogeneity. As seven out of the eight included studies were based on previous medical history and the other one study was included based on random blood glucose (≥11.1 mmol/l), there is a potential reporting bias. The result of the recalculation for the seven studies that were based on previous medical history of diabetes mellitus was still similar (OR: 1.68; 95% CI: 1.02–2.75; *P* < 0.05; *I*^2^ = 45%).

#### Obesity

In our study, we performed subgroup analyses according to WHO-recommended cutoff points for BMI and defined overweight as BMI 25 to 29.9 kg/m^2^ and obesity as BMI ≥ 30 kg/m^2^ (39). For the assessment of obesity as a potential risk factor, four studies with 629 patients were included. The result shows that obesity is a risk factor for cardiotoxicity (OR: 1.72; 95% CI: 1.13–2.61; *P* = 0.010; *I*^2^ = 73%; Figure 4). Overweight was not associated with cardiotoxicity (OR: 1.08; 95% CI: 0.65–1.80; *P* = 0.770; Supplementary Figure 1).

#### Hypercholesterinemia

For the assessment of hypercholesterinemia as a potential risk factor, five studies with 5,789 patients were included. No significant association was found between hypercholesterinemia and AIC events (OR: 1.48; 95% CI: 0.99–2.20; *P* > 0.05; *I*^2^ = 29%; Supplementary Figure 2).

#### Smoking

For the assessment of smoking as a potential risk factor, nine studies with 5,862 patients were included. No significant association was found between smoking and AIC events (OR: 1.62; 95% CI: 0.94–2.77; *P* > 0.05; *I*^2^ = 69%; Supplementary Figure 2).

### GLS in the Detection of AIC

**Figure 5:**
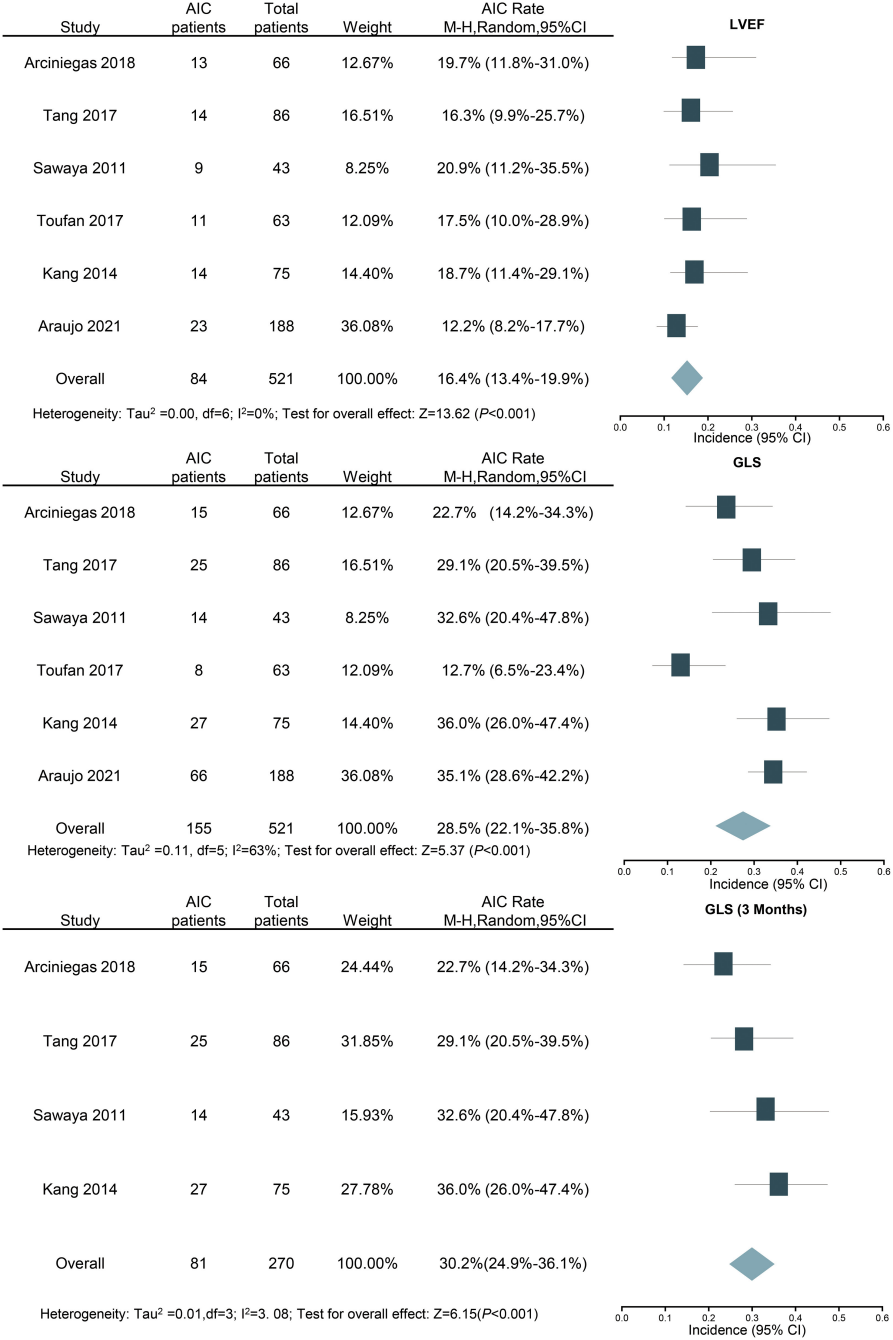
Forest plot for the incidence of anthracycline-induced cardiotoxicity by LVEF and GLS (overall and 3 months). CI, confidence intervals; GLS, global longitudinal strain; LVEF, left ventricular ejection fraction.

In the six studies examining GLS, 155 out of the 521 patients were identified as cardiotoxicity. The total incidence was 28.5, and 95% CI was 22.1 to 35.8% (*I*^2^ = 63%, *P* < 0.001, Figure 5). In sensitivity analysis, the result was still similar when the study with the largest number of patients was excluded (26.5%; 95% CI: 19.3–35.2%; *P* < 0.001). Furthermore, the above studies were also analyzed using LVEF, and the incidence of cardiotoxicity was 16.4% (84 out of the 521 patients), and 95% CI ranged from 13.4 to 19.9% (*I*^2^ = 0%, *P* < 0.001, Figure 5). For further detailed analysis, we consulted a review and meta-analysis of the literature published in 2019, and it showed that the inclusion of strain parameters in echocardiographic evaluation during cancer treatment may help to detect cardiac change earlier (40). Therefore, the detection time was taken as the grouping condition for subgroup analysis. Four studies examined the cardiotoxicity just after 3 months of chemotherapy; the incidence of AIC according to GLS was 30.2% (81 out of the 270 patients), and 95% CI was 24.9% to 36.1% (*I*^2^ = 7.70%, *P* < 0.001, Figure 5).

### Publication Bias

**Figure 6:**
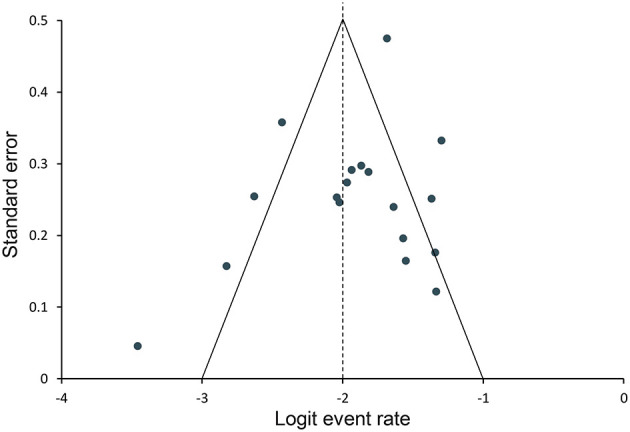
Funnel plot for assessing publication bias. Data points represent individual studies. The *y*-axis represents the measurement of study precision (plotted as standard error of effect size), and the *x*-axis represents point estimates for each study. Dashed triangular lines represent the region in which 95% of studies are expected to lie in the absence of bias and heterogeneity.

The funnel plots for all studies are combined in Figure 6. No evidence of publication bias was identified.

## Discussion

**Figure 7:**
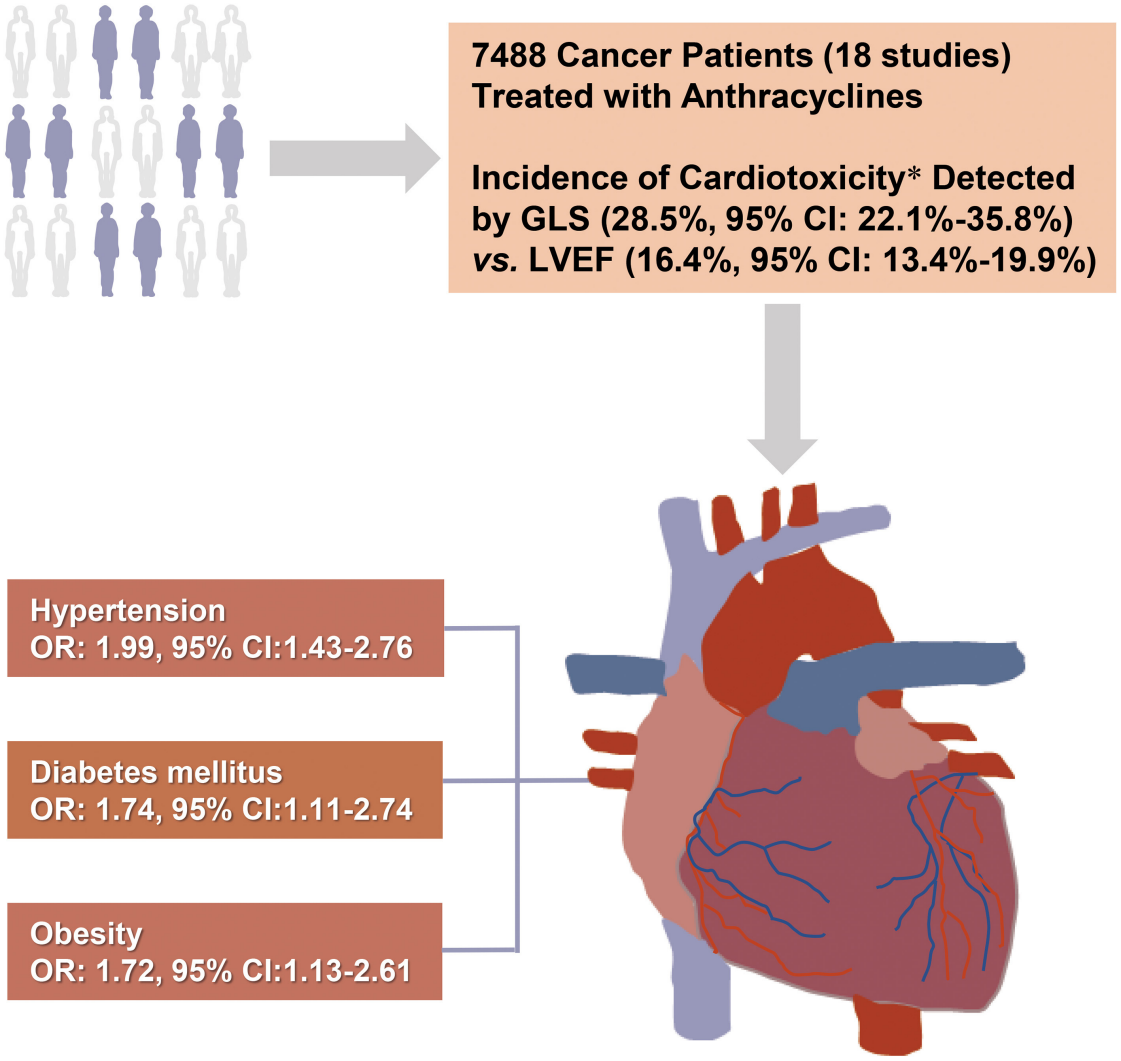
Central illustration: Risk factors for anthracycline-induced cardiotoxicity. The present systematic review included 18 studies of 7,488 cancer patients receiving anthracycline chemotherapy. Hypertension, diabetes, and obesity were identified as risk factors for anthracycline-induced cardiotoxicity. GLS showed higher detection rate and better early detection ability for cardiotoxicity than LVEF. *6 of the 18 studies used both LVEF and GLS. CI, confidential interval; LVEF, left ventricular ejection fraction; GLS, global longitudinal strain.

In this large-scale meta-analysis, we calculated the total incidence of cardiotoxicity in cancer patients receiving anthracycline chemotherapy and assessed the relationship between potential risk factors and cardiotoxicity. Among the survivors of cancer chemotherapy, hypertension (OR: 1.99; 95% CI: 1.43–2.76), diabetes mellitus (OR: 1.74; 95% CI: 1.11–2.74), and obesity (OR: 1.72; 95% CI: 1.13–2.61) were revealed to be risk factors for AIC (Figure 7). GLS showed better detection ability than LVEF, which can increase the detection rate of cardiotoxicity in the early stage (3 months after chemotherapy).

### Incidence of AIC

In a recent review, it was shown that 9% of people receiving anthracycline chemotherapy developed cardiotoxicity, and cardiotoxicity occurred within the first year after the completion of chemotherapy in 98% of cases (41). Our results were largely in agreement with those data, but with obvious heterogeneity, which was mainly attributed to the different cancer types and corresponding treatment options. There are 12 studies that only study breast cancer, and the remaining six studies include breast cancer, sarcoma, lymphoma, bladder cancer, and some leukemias. Another source of the heterogeneity is the different follow-up time. It has been reported that the cumulative incidence of cardiotoxicity increases with time (42). The follow-up time in the included studies ranged from 1 to 10 years. Twelve (66.7%) studies had a follow-up period less than 2 years.

### The Risk Factors With AIC

The link between hypertension and AIC was first proposed in 1979. Current cellular and molecular research suggests that the pathophysiological mechanism of hypertension on myocardial cell damage in AIC patients may be related to oxidative stress and inflammatory response of cardiomyocyte fibrosis (43). Although hypertension has been suggested as a risk factor for cardiotoxicity, the existing evidence has not yet reached agreement (44, 45). The limitations of previous studies included small sample size and the confounding cardioprotective drugs for hypertension. Therefore, we conducted this meta-analysis with 7,815 patients in 16 retrospective studies. Furthermore, the latest review confirmed that various anti-hypertension drugs, especially β-blockers (carvedilol and nebivolol), have shown a preventive effect for AIC (46). Thus, we excluded the studies reporting any history of these anti-hypertension drugs, which improved the quality of our report.

The exposure of the heart to the environment of diabetes and high blood sugar (including fatty acids and triglycerides) will increase fatty acids and cytokines, leading to the continuous accumulation of fat droplets in myocardial cells and ultimately mediating cardiotoxicity (47). In our research, it is further confirmed that diabetes is a risk factor for cardiotoxicity, which is consistent with previous reports (48). However, previous researches rarely classified diabetes types, which further ignored the influence of different diabetes treatment regimens (49). Therefore, the present results still need confirmation by multi-center large-scale randomized controlled studies.

Several animal experiments have shown that high-fat-diet-induced obese or overweight rats demonstrated increased sensitivity to AIC (50, 51). It may be related to increased cardiac oxidative stress and metabolic changes, such as increased leptin and decreased adiponectin levels (52, 53). A recent meta-analysis showed that obesity and overweight in breast cancer patients receiving anthracycline chemotherapy increase the risk of cardiotoxicity (39). Our results only agreed that obesity is a risk factor for AIC. Being overweight seems not a risk factor for AIC. The main reason for the discrepancy was that previous studies did not separate obesity from overweight, leading to an overlap in the analysis. However, the potential negative effect of overweight on the cardiovascular system in cancer patients should still be alerted.

Hypercholesterolemia and other metabolic stresses lead to endothelial dysfunction and vascular complications (54), which can cause cardiotoxicity in patients undergoing chemotherapy. Although it seems that hypercholesterolemia might be related to increased risk of cardiotoxicity as reported in some studies (27, 29, 55), our analysis found no obvious association between hypercholesterolemia and AIC. The various definitions of hypercholesterolemia and limited number of related studies might account for the indeterminate results.

Smoking is known to induce thrombosis and cardiovascular toxicity through inflammation and oxidative stress (56, 57). However, current data cannot confirm the relationship between smoking and subsequent cardiotoxicity in patients with anthracycline chemotherapy. Smoking habit can be very different among patients, regarding the numbers of cigarettes, frequency of smoking, number of years smoking, and other detailed items. Therefore, it is hard to draw the conclusion without a specialized and comprehensive study design, as smoking is always reported as yes or no in AIC studies.

### Advantages of GLS in the Detection of AIC

AIC mainly leads to cardiomyocyte apoptosis and myofilament degradation and thus to damage of myocardial deformation (46). Many studies have shown that GLS was superior to LVEF in the detection of early and subclinical myocardial dysfunction, with good repeatability (32, 58, 59). The diagnostic and prognostic values of GLS for cardiotoxicity have been validated by previous original researches and meta-analysis (14, 60, 61), with LVEF as the reference method. GLS has also been recommended as an early diagnostic parameter for cardiotoxicity in ESC Cardio-Oncology position statements (18, 19). In the present study, a pathologically reduced GLS was found when LVEF was still preserved in a significant fraction of patients. On the other hand, the incidence of AIC diagnosed by GLS just after 3 months of chemotherapy was quite similar with the overall detection rate during the follow-up, while the detection rate by LVEF increased with the extension of follow-up period until 5 to 10 years. All these findings provided additional proofs for the advantages of GLS in detection of AIC.

### Study Limitations

The current analysis has some limitations worth discussing. First, the number of studies included in each risk factor group was limited. Nevertheless, the publication bias assessment and sensitivity analysis showed that our results are reassuring. Second, the median age of patients in 13 of 18 studies was over 50 years old, causing a bias with lack of the young adult patients. The effects of these risk factors identified in the present studies on young adult cancer patients merit further investigation. Third, the current study did not propose a dose-dependent relationship between the risk factors and the incidence of cardiotoxicity. It was because the data we extracted are dichotomous rather than continuous variables. In addition, although most of the included studies (13 out of 18) followed the definitions of cardiotoxicity by ESC Cardio-Oncology position statements (reduction of LVEF to below 50% or a >10% reduction from baseline) (13, 18, 19), some of the early literatures did not meet the cut-offs identically. The major reason is that those studies were performed before the uniform cut-off was proposed. We removed the five studies using different cut-offs and got similar overall incidence of cardiotoxicity (14.1%, 95% CI: 10.1 to 19.4%).

## Conclusions

Our research has determined hypertension, diabetes mellitus, and obesity as risk factors for AIC, causing nearly double morbidity of cardiotoxicity. Careful attention should be paid to patients with these risks, and corresponding protective strategies should be used during and after anthracycline treatment. The higher detection rate and better early detection ability of cardiotoxicity than LVEF added new proofs for the advantages of GLS in detection of AIC.

## Data Availability Statement

The original contributions presented in the study are included in the article/Supplementary Material, further inquiries can be directed to th corresponding author/s.

## Author Contributions

SQ and TZ developed the search strategy and wrote the manuscript. BQ, YZha, YZho, and HY checked the search and reviewed the manuscript. JZ and LL performed literature screening and data extraction and conducted the quality assessment of the included studies. LY and GY carried out the data analysis. YD and CX designed the project, reviewed the manuscript, and finally approved the version to be published. All authors contributed to the article and approved the submitted version.

## Funding

This study was supported by grants from the National Natural Science Foundation of China (No. 81901751). CX was also supported by the Eyas Program of the Air Force Medical University.

## Conflict of Interest

The authors declare that the research was conducted in the absence of any commercial or financial relationships that could be construed as a potential conflict of interest.

## Publisher's Note

All claims expressed in this article are solely those of the authors and do not necessarily represent those of their affiliated organizations, or those of the publisher, the editors and the reviewers. Any product that may be evaluated in this article, or claim that may be made by its manufacturer, is not guaranteed or endorsed by the publisher.

## Abbreviations

AIC: anthracycline-induced cardiotoxicity
GLS: global longitudinal strain
LVEF: left ventricular ejection fraction
STE: speckle tracking echocardiography
2D: two-dimensional
QUADAS-2: Quality Assessment of Diagnostic Accuracy Studies.
