# Supplementary material for: Risk Factors for Anthracycline-Induced Cardiotoxicity
Source: Front Cardiovasc Med. 2021 Sep 29;8:736854. doi: 10.3389/fcvm.2021.736854 (PMC8511483; doi:10.3389/fcvm.2021.736854)
Supplement: Supplementary file 1 [file Data_Sheet_1.PDF]

Supplemental Material

Supplemental Table 1: PRISMA Checklist

Section/topic	#	Checklist item	Reported on page #
**TITLE**			
Title	1	Identify the report as a systematic review, meta-analysis, or both.	1
**ABSTRACT**			
Structured summary	2	Provide a structured summary including, as applicable: background; objectives; data sources; study eligibility criteria, participants, and interventions; study appraisal and synthesis methods; results; limitations; conclusions and implications of key findings; systematic review registration number.	2
**INTRODUCTION**			
Rationale	3	Describe the rationale for the review in the context of what is already known.	2
Objectives	4	Provide an explicit statement of questions being addressed with reference to participants, interventions, comparisons, outcomes, and study design (PICOS).	3
**METHODS**			
Protocol and registration	5	Indicate if a review protocol exists, if and where it can be accessed (e.g., Web address), and, if available, provide registration information including registration number.	N/A
Eligibility criteria	6	Specify study characteristics (e.g., PICOS, length of follow-up) and report characteristics (e.g., years considered, language, publication status) used as criteria for eligibility, giving rationale.	3
Information sources	7	Describe all information sources (e.g., databases with dates of coverage, contact with study authors to identify additional studies) in the search and date last searched.	3
Search	8	Present full electronic search strategy for at least one database, including any limits used, such that it could be repeated.	Supplemental material

Study selection	9	State the process for selecting studies (i.e., screening, eligibility, included in systematic review, and, if applicable, included in the meta-analysis).	3, Figure 1
Data collection process	10	Describe method of data extraction from reports (e.g., piloted forms, independently, in duplicate) and any processes for obtaining and confirming data from investigators.	4
Data items	11	List and define all variables for which data were sought (e.g., PICOS, funding sources) and any assumptions and simplifications made.	4
Risk of bias in individual studies	12	Describe methods used for assessing risk of bias of individual studies (including specification of whether this was done at the study or outcome level), and how this information is to be used in any data synthesis.	4, Figure 2, Supplemental material
Summary measures	13	State the principal summary measures (e.g., risk ratio, difference in means).	4
Synthesis of results	14	Describe the methods of handling data and combining results of studies, if done, including measures of consistency (e.g., I2) for each meta-analysis.	4
Risk of bias across studies	15	Specify any assessment of risk of bias that may affect the cumulative evidence (e.g., publication bias, selective reporting within studies).	4
Additional analyses	16	Describe methods of additional analyses (e.g., sensitivity or subgroup analyses, meta-regression), if done, indicating which were pre-specified.	4
**RESULTS**			
Study selection	17	Give numbers of studies screened, assessed for eligibility, and included in the review, with reasons for exclusions at each stage, ideally with a flow diagram.	5, Figure 1
Study characteristics	18	For each study, present characteristics for which data were extracted (e.g., study size, PICOS, follow-up period) and provide the citations.	Table 1
Risk of bias within studies	19	Present data on risk of bias of each study and, if available, any outcome level assessment (see item 12).	Supplemental material

Results of individual studies	20	For all outcomes considered (benefits or harms), present, for each study: (a) simple summary data for each intervention group (b) effect estimates and confidence intervals, ideally with a forest plot.	5-6
Synthesis of results	21	Present results of each meta-analysis done, including confidence intervals and measures of consistency.	Figure 3-5
Risk of bias across studies	22	Present results of any assessment of risk of bias across studies (see Item 15).	6, Figure 6
**DISCUSSION**			
Summary of evidence	24	Summarize the main findings including the strength of evidence for each main outcome; consider their relevance to key groups (e.g., healthcare providers, users, and policy makers).	7-9
Limitations	25	Discuss limitations at study and outcome level (e.g., risk of bias), and at review-level (e.g., incomplete retrieval of identified research, reporting bias).	9
Conclusions	26	Provide a general interpretation of the results in the context of other evidence, and implications for future research.	9
**FUNDING**			
Funding	27	Describe sources of funding for the systematic review and other support (e.g., supply of data); role of funders for the systematic review.	9

Supplemental Table 2: Revised Quality Assessment of Diagnostic Accuracy Studies (QUADAS-2) scores for included studies.

Studies	Risk of Bias				Applicability Concerns		
	Patient Selection	Index Text	Reference Standard	Flow and Timing	Patient Selection	Index Text	Reference Standard
Vaitiekus 2020	Low	Low	High	Low	Low	Low	Unclear
Chung 2013	Low	Low	Unclear	Low	Low	Low	Low
Cho 2020	Low	Low	Unclear	High	Low	Low	Unclear
Hequet 2004	Low	Low	Low	High	Low	Low	Low
Wang 2015	Low	Low	Low	Low	Low	Low	Low
Araujo 2021	High	Low	Unclear	Low	Low	Low	Unclear
Piotrowski 2012	Low	Low	Unclear	Low	Low	Low	Low
Gunaldi 2016	Low	Low	Low	Low	Low	Low	Low
Cardinale 2015	Low	Low	Low	High	Low	Low	Low
Toufan 2017	Low	Low	Low	Low	Low	Low	Low
Kang 2014	Low	Low	Low	Low	Low	Low	Low
Sawaya 2011	High	Low	Unclear	Low	Low	Low	Unclear
Tang 2017	Low	Low	Unclear	Low	Low	Low	Unclear
Arciniegas 2018	Low	Low	Low	Low	Low	Low	Low
Aitelhaj 2013	Low	Low	Unclear	Low	Low	Low	Low
Caram 2015	Low	Low	Low	Low	Low	Low	Low
Karanth 2011	Low	Low	Unclear	High	Low	Low	Low
Mina 2015	Low	Low	Low	High	Low	Low	Low

Supplemental Figure 1. Risk of bias for included studies

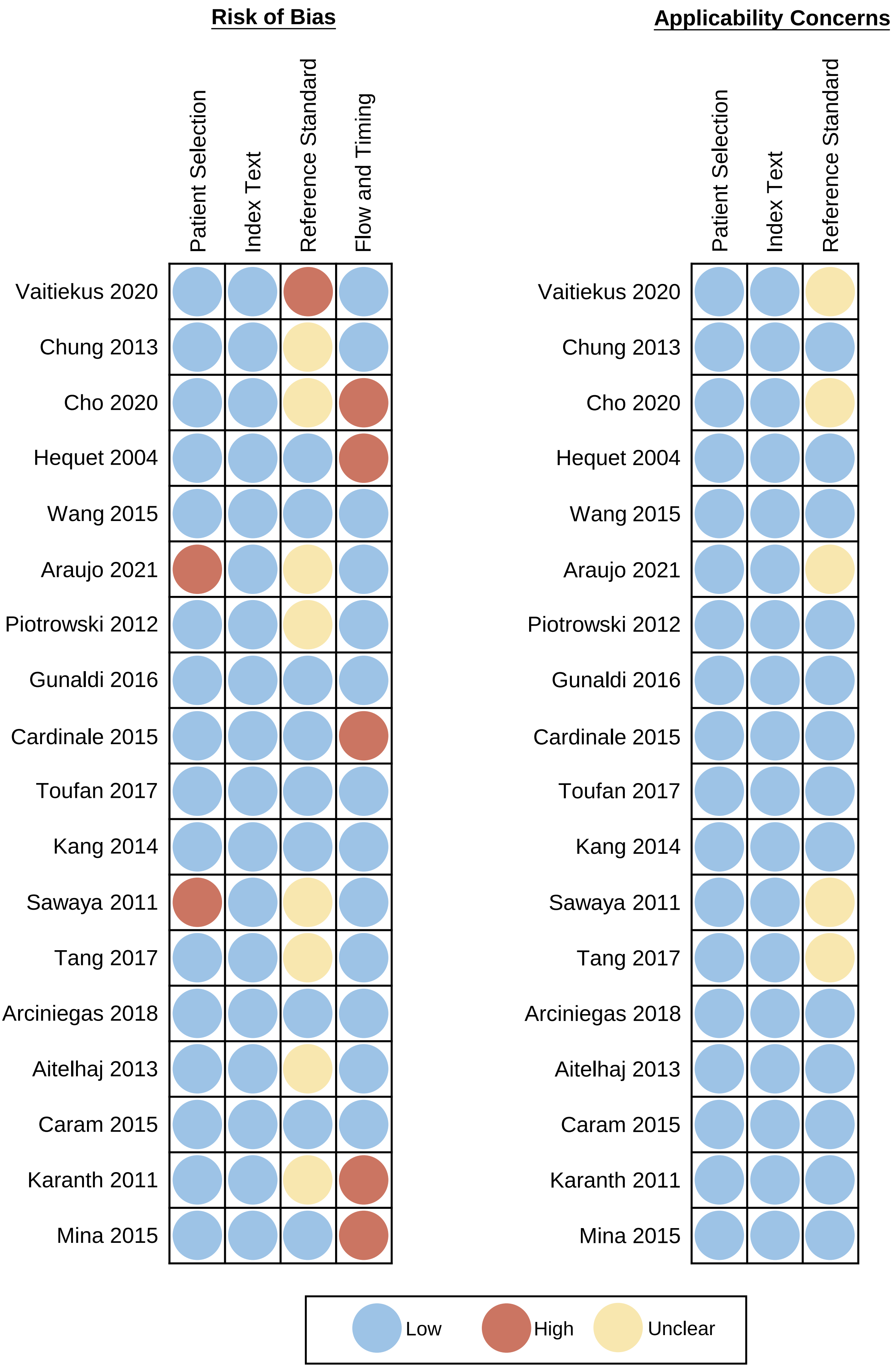

The methodological quality of included studies was assessed with revised Quality Assessment of Diagnostic Accuracy Studies (QUADAS-2) tool. This tool comprises 4 domains: patient selection, index test, reference standard, and flow and timing. Each domain is assessed in terms of risk of bias, and the first 3 domains are also assessed in terms of concerns regarding applicability.

Supplemental Figure 2. Forest plot of the relationship between smoking, hypercholesterolemia and overweight and anthracycline-induced cardiotoxicity

Study	AIC Patients	AIC Total	No AIC Patients	No AIC Total	Weight	OR M-H, Random, 95%CI
Piotrowski 2012	7	52	27	201	12.0%	1.00 (0.41-2.45)
Cardinale 2015	36	226	483	2399	16.5%	0.75 (0.52-1.09)
Kang 2014	5	14	17	61	9.3%	1.44 (0.42-4.91)
Araujo 2021	15	23	33	165	11.6%	7.50 (2.93-19.18)
Hequet 2004	10	68	8	73	11.1%	1.40 (0.52-3.79)
Karanth 2011	16	29	62	188	12.9%	2.50 (1.13-5.52)
Wang 2015	5	65	61	2220	10.7%	2.32 (0.82-6.59)
Cho 2020	6	92	27	521	11.8%	1.28 (0.51-3.18)
Sawaya 2011	1	9	5	34	4.2%	0.72 (0.07-7.13)
Overall	101	578	723	5862	100%	1.62 (0.94-2.77)

Heterogeneity: Tau^2 =0.42; Chi^2=26.15, df=8; I^2=69%; Test for overall effect: Z=1.75 (*P*=0.08)

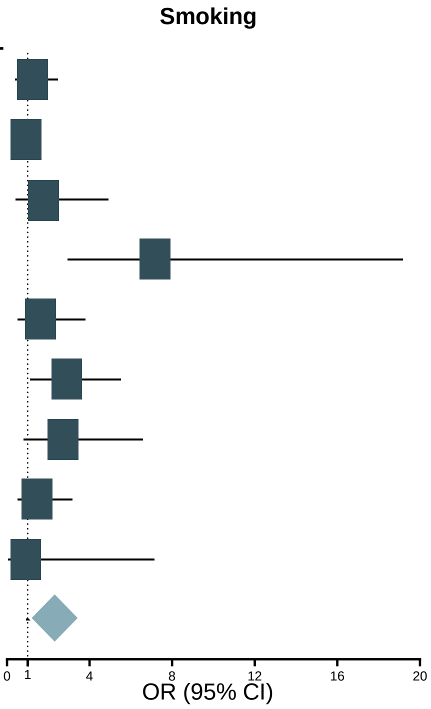

Study	AIC Patients	AIC Total	No AIC Patients	No AIC Total	Weight	OR M-H, Random, 95%CI
Piotrowski 2012	17	52	70	201	24.6%	0.91 (0.48-1.74)
Cardinale 2015	20	226	142	2399	34.0%	1.54 (0.95-2.52)
Araujo 2021	8	23	44	165	14.7%	1.47 (0.58-3.70)
Hequet 2004	14	68	4	73	10.1%	4.47 (1.39-14.36)
Wang 2015	6	65	149	2220	16.6%	1.41 (0.60-3.33)
Overall	65	434	382	5789	100%	1.48 (0.99-2.20)

Heterogeneity: Tau^2 =0.06; Chi^2=5.65, df=4; I^2=29%; Test for overall effect: Z=1.90 (*P*=0.06)

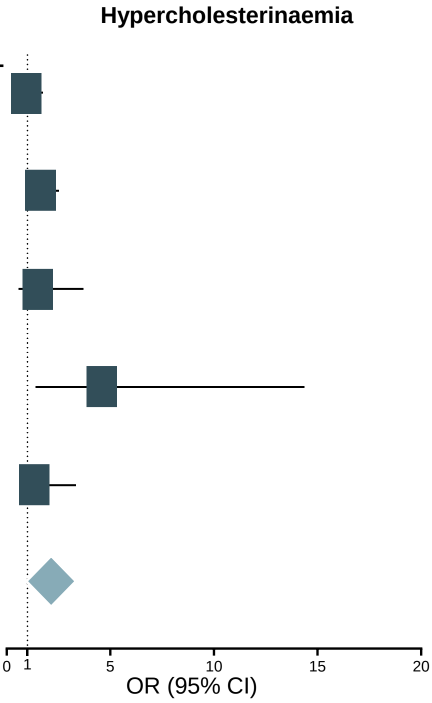

Study	AIC Patients	AIC Total	No AIC Patients	No AIC Total	Weight	OR M-H, Random, 95%CI
Caram 2015	7	19	34	146	25.7%	1.92 (0.70-5.27)
Mina 2015	2	6	72	214	8.8%	0.99 (0.18-5.51)
Piotrowski 2012	19	52	80	201	65.5%	0.87 (0.46-1.64)
Overall	27	77	186	561	100%	1.08 (0.65-1.80)

Heterogeneity: Tau^2 =0.00; Chi^2=1.72, df=2; I^2=0%; Test for overall effect: Z=0.29 (*P*=0.77)

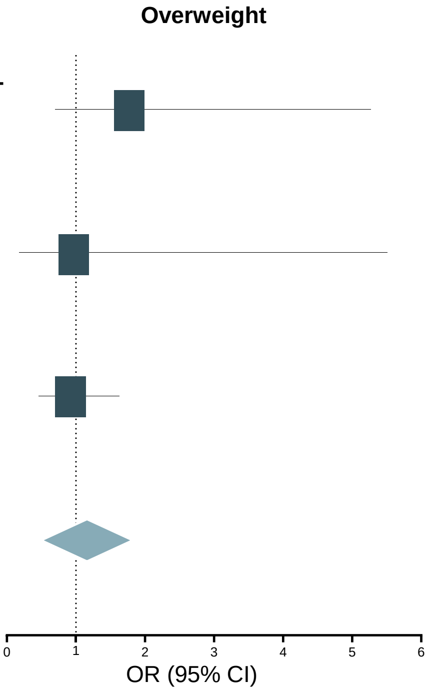

Search Strategy for Literature Review

PubMed, Embase and Cochrane Library search as run:

(((cardiotoxicity[Title/Abstract]) OR (cardiomyopathy[Title/Abstract])) AND ((((anthracycline[Title/Abstract]) OR (daunorubicin[Title/Abstract])) OR (doxorubicin[Title/Abstract])) OR (Epirubicin[Title/Abstract]))) AND ((((((risk factors[Title/Abstract]) OR (hypertension[Title/Abstract])) OR (diabetes mellitus[Title/Abstract])) OR (obesity[Title/Abstract])) OR (overweight[Title/Abstract])) OR ((((Hypercholesterolemia[Title/Abstract]) OR (Hyperlipoproteinemias[Title/Abstract])) OR (Hyperlipidemia[Title/Abstract])) OR (smoke[Title/Abstract])))

1. (cardiomyopathy [Title/Abstract]) OR (cardiotoxicity [Title/Abstract])
2. (((anthracycline[Title/Abstract]) OR (daunorubicin[Title/Abstract])) OR (doxorubicin[Title/Abstract])) OR (Epirubicin[Title/Abstract])
3. Search #1 AND Search #2
4. ((((risk factors[Title/Abstract]) OR (hypertension[Title/Abstract])) OR (diabetes mellitus[Title/Abstract])) OR (obesity[Title/Abstract])) OR (overweight[Title/Abstract])
5. (((Hypercholesterolemia[Title/Abstract]) OR (Hyperlipoproteinemias[Title/Abstract])) OR (Hyperlipidemia[Title/Abstract])) OR (smoke[Title/Abstract])
6. Search #4 OR Search #5
7. Search #3 AND Search #6
